# Assessment of genetic referrals and outcomes for women with triple negative breast cancer in regional cancer centres in Australia

**DOI:** 10.1186/s13053-021-00176-z

**Published:** 2021-02-26

**Authors:** Lucie G. Hallenstein, Carol Sorensen, Lorraine Hodgson, Shelly Wen, Justin Westhuyzen, Carmen Hansen, Andrew T. J. Last, Julan V. Amalaseelan, Shehnarz Salindera, William Ross, Allan D. Spigelman, Thomas P. Shakespeare, Noel J. Aherne

**Affiliations:** 1 Cancer Genetics Service, Mid North Coast Cancer Institute, Coffs Harbour, New South Wales Australia; 2 Department of Radiation Oncology, Mid North Coast Cancer Institute, Coffs Harbour, New South Wales Australia; 3 Cancer Genetics Service, Mid North Coast Cancer Institute, Port Macquarie, New South Wales Australia; 4 Kingscliff Community Health, Kingscliff, New South Wales Australia; 5 Department of Radiation Oncology, Mid North Coast Cancer Institute, Port Macquarie, New South Wales Australia; 6 Department of Radiation Oncology, North Coast Cancer Institute, Lismore, New South Wales Australia; 7 Department of Surgery, University of New South Wales, St Vincent’s Clinical School, Sydney, New South Wales Australia; 8 Rural Clinical School Faculty of Medicine, University of New South Wales, St Vincent’s Clinical School, Sydney, New South Wales Australia; 9 Cancer Genetics Unit, The Kinghorn Cancer Centre, St Vincent’s Hospital, Sydney, New South Wales Australia; 10 School of Health and Human Sciences, Southern Cross University, Coffs Harbour, New South Wales Australia

**Keywords:** Breast cancer, Triple negative, Genetic counselling, BRCA, Hereditary cancer, Regional, Panel testing, Re-contacting

## Abstract

**Background:**

Guidelines for referral to cancer genetics service for women diagnosed with triple negative breast cancer have changed over time. This study was conducted to assess the changing referral patterns and outcomes for women diagnosed with triple negative breast cancer across three regional cancer centres during the years 2014–2018.

**Methods:**

Following ethical approval, a retrospective electronic medical record review was performed to identify those women diagnosed with triple negative breast cancer, and whether they were referred to a genetics service and if so, the outcome of that genetics assessment and/or genetic testing.

**Results:**

There were 2441 women with newly diagnosed breast cancer seen at our cancer services during the years 2014–2018, of whom 237 women were diagnosed with triple negative breast cancer. Based on age of diagnosis criteria alone, 13% (31/237) of our cohort fulfilled criteria for genetic testing, with 81% (25/31) being referred to a cancer genetics service. Of this group 68% (21/31) were referred to genetics services within our regions and went on to have genetic testing with 10 pathogenic variants identified; 5x *BRCA1*, 4x *BRCA2* and × 1 *ATM*:c.7271 T > G.

**Conclusions:**

Referral pathways for women diagnosed with TNBC to cancer genetics services are performing well across our cancer centres. We identified a group of women who did not meet eligibility criteria for referral at their time of diagnosis, but would now be eligible, as guidelines have changed. The use of cross-discipline retrospective data reviews is a useful tool to identify patients who could benefit from being re-contacted over time for an updated cancer genetics assessment.

## Background

Breast cancer is the most commonly diagnosed cancer in women between the ages of 30 and 80 years in Australia, affecting one in seven women by age 85 [1]. Women diagnosed with triple negative breast cancer (TNBC: oestrogen, progesterone and human epidermal growth factor receptor 2 [HER2] negative) comprise 10–15% of all breast cancer diagnoses [2] and are more likely to carry a germline *BRCA1* pathogenic variant (PV) than hormone positive breast cancers [3–5]. *BRCA2* PVs are also associated with TNBC [3–5]. Women with TNBC diagnosed under 50 years have the highest likelihood of carrying a *BRCA1* PV, irrespective of family history [3, 6] and may have inferior oncological outcomes to patients who have receptor positive breast cancers.

Australian referral guidelines for consideration of publicly funded genetic testing have evolved to reflect the changing knowledge of familial cancer, particularly for women diagnosed with TNBC. In Australia, EviQ referral guidelines developed by Cancer Institute New South Wales [7], were established in 2010 for breast cancer risk assessment. These guidelines recommended all women diagnosed with TNBC aged 40 years and under be referred to a genetics service for assessment/genetic testing, irrespective of their family history. These guidelines were later revised in 2016 to all women diagnosed with TNBC at or below age 50 years [7]. Women diagnosed with TNBC over 50 years who have a family history of breast, non-mucinous epithelial ovarian, fallopian tube or primary peritoneal cancer in a close relative, are also recommended to be referred to a genetics service for assessment and genetic testing if appropriate [7]. This is summarised in Table 1.

**Table 1:** Changes over time to referral guidelines for women diagnosed with TNBC

Characteristics that warrant referral to genetics service	2010 referral guidelines	2016 referral guidelines
**Tumour pathology**	TNBC diagnosed 40 years and under	TNBC diagnosed 50 years and under
**Family history**	Breast, non-mucinous epithelial ovarian, fallopian tube or primary peritoneal cancer	Breast, non-mucinous epithelial ovarian, fallopian tube or primary peritoneal cancer

In addition to changes in genetic testing referral guidelines, the approach to genetic testing has evolved from single gene testing to the use of breast cancer panels. In Australia, in addition to *BRCA1/*2 genes, typical breast cancer panels include *PALB2, ATM* (c.7271 T > G), *TP53, CHEK2* (c.1100delC). We note that some of these genes (e.g. CHEK2 [c.1100delC]) are not typically associated with TNBC, but are commonly included in breast cancer panels in Australia. The addition of other breast/ovarian cancer genes is available if patients meet testing criteria (e.g. *CDH1*) or have a relevant personal or family history. Advances in technology have greatly improved the utility of genetic testing and reduced costs, allowing for more comprehensive genetic testing.

Timely identification of individuals with a breast cancer gene pathogenic variant is important as it may help inform treatment options, including targeted therapy and involvement in clinical trials [8–10]. It also allows for subsequent implementation of risk-reducing and/or early detection strategies; for example, *BRCA1/2* PV carriers have an increased risk of developing contralateral breast cancer (40% for *BRCA1* and 26% for *BRCA2* at 20 years after initial diagnosis) [10] and ovarian cancer (lifetime risk of 44% for *BRCA1* and 17% *BRCA2*) [10]. The risk of pancreatic cancer is also increased (< 5%) in individuals with a *BRCA*2 PV [11, 12]. As well as being beneficial for the individual, identification of a PV allows for at-risk biological relatives to access predictive testing to inform their own cancer risk and appropriate risk management.

Our centres comprise three regional cancer centres located in regional New South Wales. All women newly diagnosed with breast cancer are discussed in weekly breast multidisciplinary team (MDT) meetings in each respective centre. Members of the Breast MDT meeting include representation from genetic counselling, medical oncology, radiation oncology, surgery, clinical trials, pathology, radiology, allied health and breast care nursing. Genetic counsellors assess and identify individuals who would be appropriate to refer to genetics services for review of personal and family history, with a view to providing a risk assessment and genetic testing where suitable. Each site has an associated cancer genetics service with a local genetic counsellor. Having genetic counsellors onsite providing high-quality services in our regional areas removes many barriers of care and has a positive impact for our patients. Despite the increasing patient load over time, the cancer genetic services remain resourced with one genetic counsellor at each service, two of whom also cover general genetics and one who is employed half-time for cancer genetics only. Each genetic counsellor works in tandem with a single consultant cancer geneticist. Treatment and survival outcomes for women with TNBC at our centres has been published previously [13].

This study was conducted to provide an insight into referral rates and outcomes for women diagnosed with TNBC at three regional cancer centres, which has not to our knowledge been reported in the literature. There is a similar report from a major metropolitan cancer centre however [14]. By assessing the quality of service delivered across our sites, we hope to identify areas for improvement to patient care, which we think will be transferable to other services.

## Methods

### Study design and population

This is a retrospective electronic medical record review, examining breast MDT referrals to genetics services for all female patients diagnosed with TNBC at our cancer centres, between 2014 and 2018 inclusive. This paper assesses whether a referral was made, as well as, the subsequent outcome of genetic assessment and testing for women diagnosed with TNBC at our cancer services. This study was reviewed by North Coast NSW Human Research Ethics Committee (NCNSW HREC QA346) and was considered a quality improvement project.

### Data collection

Data searches were performed in the Mosaiq electronic medical record (Elekta, Crawley, United Kingdom) to identify women diagnosed with TNBC at our cancer centres in the years 2014–2018 inclusive. Data were extracted, providing information as to receptor status, age and year diagnosed. Women without complete receptor status were included in the initial search and then records manually examined, including women in the data set where TNBC could be confirmed and excluding women without TNBC or if unable to verify receptor status.

A search of women diagnosed with breast cancer was then performed in the multi-state New South Wales and Australian Capital Territory state-wide genetic database (Trakgene), with parameters set to only include women referred to our local genetics services. The records of women diagnosed with TNBC were extracted and the data collated to provide information regarding whether a referral was made, if they booked and/or attended an appointment, what genetic testing occurred and the outcome of that genetic testing. If a woman had genetic testing more than once, (ie. had *BRCA1/2* testing only, then later complete breast cancer panel testing), results were merged and the most comprehensive results included.

The separate data sets from Mosaiq (clinical outcomes) and Trakgene (genetic testing outcomes) were then merged and examined. Women in the data set without referral information were cross-checked again in a search of the Trakgene genetic database to determine if they had been referred to a genetics service outside of our local area. When identified, no further information was gathered beyond recording they had been referred, as per our ethics approval. The combined datasets were then de-identified and analysed.

### Data analysis

A descriptive analysis was performed on the dataset, grouping women into age of diagnosis: women diagnosed at or below 40 years, 41–50 years and over 50 years. These parameters were used to extract information about if/when women were referred and related age guidelines applicable at the time. Women in the 41–50 year age group were divided into two groups, diagnosed before or after 2016, as this is when the EviQ guidelines changed. Information relating to referrals, whether genetic testing was offered, the type of testing was performed (single gene or panel testing) and outcomes of genetic testing were examined.

## Results

In the years 2014–2018 inclusive, there were 2441 women diagnosed with breast cancer across our three regional cancer centres. Of these, 237 women were diagnosed with TNBC, representing 9.7% of all women diagnosed, which is the cohort assessed in this study. The median age of diagnosis was 64 years (range 28–102 years).

### Women diagnosed at or below 40 years

During the period of the study (2014–2018), 14 women aged at or below 40 years were diagnosed with TNBC (6% of total dataset, 14/237). Of these women, 50% (7/14) were referred to their local genetics service and all had genetic testing, as per guidelines. The testing performed consisted of 43% (3/7) gene testing for BRCA1/2 only, 43% (3/7) breast cancer panel testing and 14% (1/14) predictive *BRCA1* test. This resulted in the detection of three pathogenic variants; 66.6% (2/3) *BRCA1* PV and 33.3% (1/3) *BRCA2* PV. There were no PVs identified in other breast cancer susceptibility genes tested for in the panel testing. Of the 50% (7/14) women not referred to a genetics service in our local areas, 14% (1/7) was referred to a genetics service outside of our catchment areas, we do not have referral information for the remaining 86% (6/7) of this group.

### Women diagnosed 41–50 years

Prior to the change in guidelines in 2016, there were 10 women between 41 and 50 years who were diagnosed with TNBC, representing 4% (10/237) of the total dataset. These women were not eligible for referral at their time of diagnosis, based only on their age. Nevertheless, 30% (3/10) of these women were referred to genetics service as they had a family history of breast, non-mucinous epithelial ovarian, fallopian tube or primary peritoneal cancer in a close relative. All went on to have genetic testing, 100% (3/3) had *BRCA1/2* testing only, and there were no breast cancer panel or predictive tests arranged. The testing resulted in one (33.3% 1/3) *BRCA2* PV being identified.

Of the women not referred to our local genetics services, 20% (2/10) were referred to an out of area genetics service. Genetic referral information is not available for the remaining women 50% (5/10).

After the guideline change in 2016, there were 17 women between 41 and 50 years diagnosed with TNBC, representing 7% (17/237) of the total dataset. Of these women, 82% (14/17) were referred to their local genetics service. All (100%, 14/14) of these women were offered genetic testing as per the guidelines, 93% (13/14) went ahead with testing and one woman (7%, 1/14) declined genetic testing. Testing comprised of 23% (3/13) *BRCA1/2* testing only and 76% (10/13) breast cancer panel testing, there were no predictive tests performed. From this testing, a single (8%, 1/13) *BRCA1* PV was identified, and a different individual was found to carry a *PALB2* variant of uncertain significance (VUS) (8%, 1/13). Of the remaining 18% (3/17) of women not referred to our local genetics services, all were referred to another genetics service out of area.

### Women diagnosed over 50 years

The 196 women who were diagnosed with TNBC aged over 50 years, represented 82% (196/237) of the total dataset. These women did not meet criteria for generic referral based on age of diagnosis. Due to a personal history of multiple cancer diagnoses, or family history of breast, non-mucinous epithelial ovarian, fallopian tube or primary peritoneal cancer in a close relative, 22% (43/196) were referred to their local genetics service. After a genetic assessment, 69% (30/43) met criteria for genetic testing, which comprised of 56.6% (17/30) breast cancer panel tests, 36.6% (11/30) *BRCA1/2* only tests and 6.6% (2/30) predictive tests. This resulted in 16.6% (5/30) of women with a PV identified; 20% (1/5) *ATM*:c.7271 T > G PV, 40% (2/5) *BRCA1* PV, 40% (2/5) *BRCA2* PV. There were also three women with VUS found; one *BRCA1* VUS and two *BRCA2* VUS. After an assessment by the genetics service, 15% (2/13) were not eligible for publicly funded genetic testing, as they had an affected relative who had already had genetic testing with no PV identified. There were also 33.3% (10/30) of the group who declined or did not attend an appointment. A small percentage (6%, 11/196) of women were referred to a genetics service outside of our area. The remaining 72% (142/196) of women were not referred as they did not meet criteria.

**Fig. 1:**
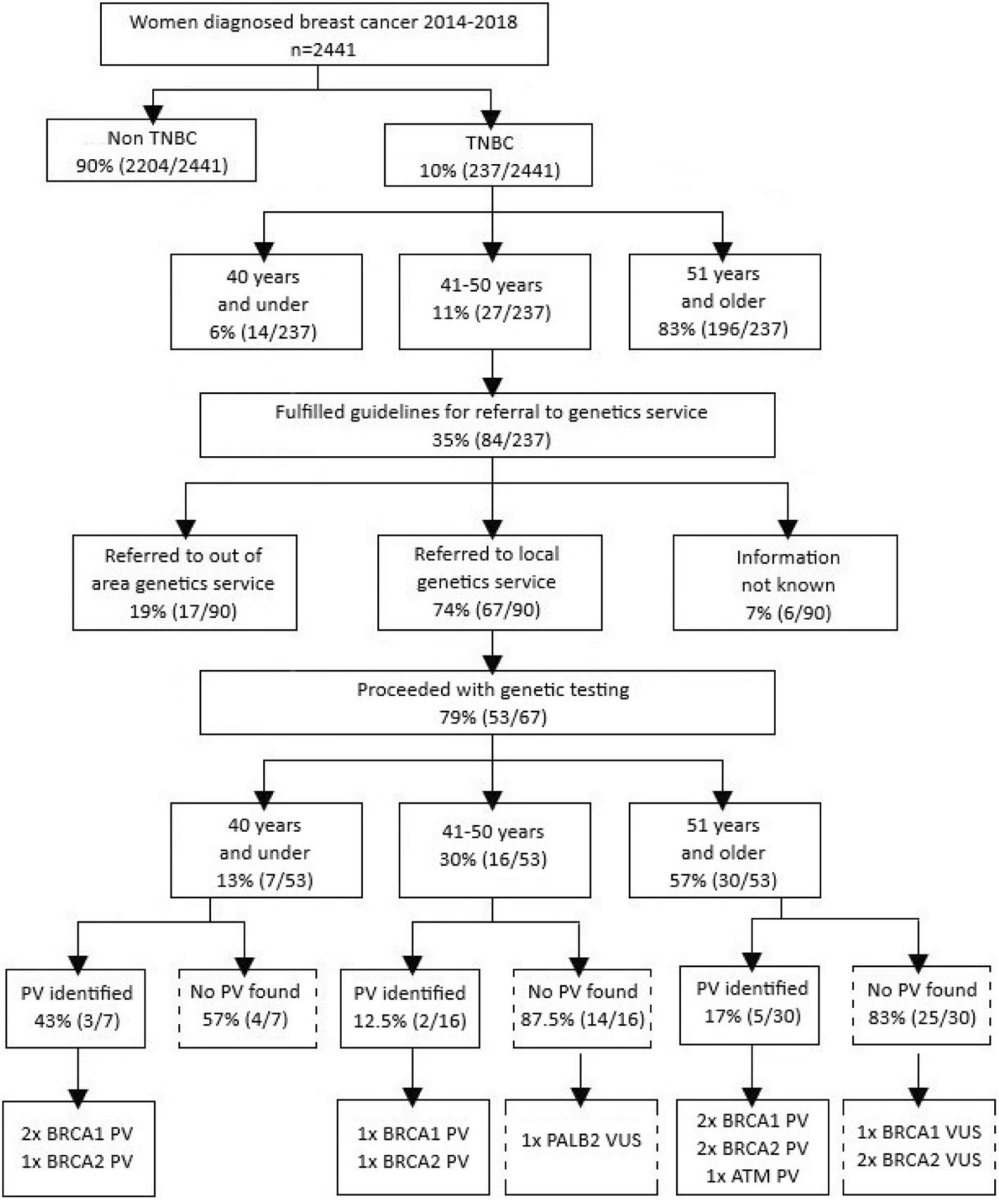
Flowchart of referral outcomes for women diagnosed with TNBC between 2014 and 2018 inclusive

In summary, there were 31 women with TNBC eligible at their time of diagnosis for referral to a genetics service based on their receptor status and age at diagnosis. This represented 13% (31/237) of the total data set. Of these women 81% (25/31) were referred to a genetics service; comprising of 68% (21/31) referred to their local genetics service and 13% (4/31) to a service outside of our local area. Of these women seen in our area, 20% (4/20) had a PV identified, comprising of 75% (3/4) *BRCA1* PV and 25% (1/4) *BRCA2* PV. There were no PVs identified in other breast cancer susceptibility genes included in panel testing. This is represented in Fig. 1.

Of the 87% (206/237) of women in the dataset who did not meet criteria for referral based purely on their age at diagnosis, 22% (46/206) were referred to a genetics service as they had multiple diagnoses of cancer or a family history of breast, non-mucinous epithelial ovarian, fallopian tube or primary peritoneal cancer in a close relative. Within this group, 13% (6/46) had a PV identified; 50% (3/6) *BRCA1* PVs, 33.3% (2/6) *BRCA2* PVs and 16.6% (1/6) *ATM*:c.7271 T > G PV. There were also 9% (4/46) who had a VUS identified, comprising of 25% (1/4) *BRCA1* VUS, 50% (2/4) *BRCA2* VUS, and 25% (1/4) *PALB2* VUS. The *BRCA1* and *BRCA2* VUS were all identified in women over age 50 years, the *PALB2* VUS was identified in a woman aged between 41 and 50 years.

## Discussion

This study reports on the referrals to cancer genetics services made by Breast MDT meetings within our three cancer centres for women diagnosed with TNBC. We have demonstrated 81% (25/31) of women who fulfilled the TNBC age criteria for genetics referral at their time of diagnosis, were appropriately referred to a genetics service for discussion of genetic testing. Of this group, 68% (21/31) were women referred within our regions. These findings compare favourably with the 58.5% (10/17) appropriately referred patients described in a study on a major metropolitan cancer centre [14]. The most prevalent PV identified in our cohort were *BRCA1* PVs, which made up 50% (5/10) of the total PVs identified, followed by *BRCA2* PV representing 40% (4/10) of the total PVs and a single non-*BRCA1/2* PV which was an *ATM*:c.7271 T > G PV, representing 10% (1/10) of PVs identified. Due to small sample size, we did not see a significant difference in BRCA1/2 PV identification.

Of our total cohort who had genetic testing, 19% (10/53) of women had a PV identified, with 9% (5/53) found to carry a *BRCA1* PV. Our non-*BRCA1/2* detection rate amongst the women who had panel testing was 3.3% (1/30). These findings are in line with other studies [4, 15–17].

It is important to note that genes included in the panel testing performed in this study were not always the same, as it was reliant on what was available and appropriate at the time of testing, rather than a static selection. Regarding non *BRCA1/*2 PVs, it has been suggested that *BARD1*, *BRIP1*, *PALB2*, and *RAD51C* PVs may be more prevalent in women diagnosed with TNBC [15, 16]. In the timeframe of this study, only *PALB2* was routinely involved in screening; however, this would not necessarily have been tested for in each person who had panel testing in this study.

Updating genetic testing for individuals with no PV identified or ‘re-contacting ‘is becoming common in clinical practice, as developments in technology and known cancer predisposition genes yield higher detection rates than previous testing available. This information may impact upon medical management for an individual at the time of their diagnosis. Various studies looking at updating genetic testing for people who previously had *BRCA1/2* testing with no PV identified (not limited to TNBC), showed a PV detection rate between 4 and 11.4% [18–21]. If genetic testing was updated for the 20 women who had BRCA1/2 testing only, it would be expected that approximately one to two women would have a PV identified. Currently updated genetic testing is performed ad-hoc in our genetics services and many others, usually prompted by a referral for the individual or their relatives.

Guidelines for genetics referral and testing are changing rapidly with the increasing knowledge in cancer genetics. Since the change in guidelines for women diagnosed with TNBC, we identified 5% (11/237) of the total cohort who would now meet criteria for genetic testing, but did not at their time of diagnosis. It is possible that some of these individuals have seen an out of area or private genetics service since the guidelines changed; we do not have access to records to verify this. There are many studies looking at re-contacting patients within a genetics context, investigating how best do this when there is updated information that may benefit the patient [22–26]. There is no consensus or procedures in place at present to guide service delivery in re-contacting patients [22–26].

It is not clear whose responsibility it is to inform patients of updated genetic information. Is it the role of genetics services, specialist clinicians, general practitioners or the patients themselves [22–25]? As per the Human Genetics Society of Australasia Clinical Genetics Service Framework [27], our services encourage patients to re-contact the service for updated information over time. Anecdotally, we find patients rarely re-contact our genetics services for updated information. Studies have shown that the majority of patients do want to be re-contacted when relevant information is available [22, 28], suggesting other methods to facilitate this would be beneficial for our patients.

Response rates to mailed letters were examined by Sawer et al. [26]. The authors sent a letter to patients who had had testing with no PV identified, suggesting they re-contact the service to have their testing updated. Seven months after sending the letter only 4.27% of people had seen the genetics service as a result of the letter. The authors experimented with four different versions of the letter, focusing on different benefits of further testing (ie. for the person themselves versus benefit to relatives), and found no differences in response rate. They deduced, that while the letter had a very low response rate, it did fulfil the service’s duty of care to notify patients of updated information [26].

Telephoning patients has also been evaluated. In a study examining contacting parents of children with intellectual disabilities to inform updated testing was available, the vast majority of parents (87%) thought re-contacting was appropriate [28]. A higher response rate was achieved, with 36% arranging a follow up appointment as result of the phone call [28]. While this was a very successful method, this is very labor intensive for a busy genetics service. It is unlikely that this would be possible for most genetics services without specific additional operational funding and resources.

Another method for updating genetic testing is via research projects. In the genetic testing consent process, patients provide consent for updating testing to be performed on their stored DNA. This is reliant on funded research projects to organise the re-testing and interpretation of results. The genetics service would then contact the patient, arrange clinical confirmation of the result and the necessary clinical follow up. Rather than spreading resources across all patients, this allows for focus on those individuals with a PV identified. However, is reliant on specific research projects occurring.

The European Society of Human Genetics has addressed these concerns, stating re-contacting should occur in the best interest of the patient [24]. Responsibility should be shared between the multidisciplinary team and the patient themselves. Resources should be provided to ensure this is sustainable within the health service, including data retention, data review and sharing of information [24]. This is especially important for regional centres where patients are located in a wide geographical area. The provision of resources, both in terms of genetic counsellors and administrative support has not kept up with the large increase in overall demand, let alone allowing for additional tasks such as outlined to occur. A professional consensus is needed to guide re-contacting patients [24].

For patients still engaged with clinical services, MDT meetings provide an opportunity for review of updated genetic information. Genetic counsellor attendance at MDT meetings has been shown to improve referrals for cancer genetics [29]. In the time frame of our study, genetic counsellors were available for consult for the Breast MDT groups, however attendance was sporadic across some of our sites, due to availability within working hours or as a result of resource constraints. Advances in knowledge and technology has made genetic testing more available and extensive for patients, without a commensurate expansion of genetic counsellor roles to manage the ever greater workload [30]. Increase in resources for genetics services to allow genetic counsellors to regularly attend MDT meetings would help ensure updated genetic information is available for patient care. Increased resources would also assist in providing dedicated time for regular quality assurance projects to identify patients who may benefit from an updated genetics review without impacting waiting times for clinical care.

Our study highlights that quality assurance projects are valuable to identify patients who may benefit from an updated genetic assessment, especially those who did not meet criteria for referral at their time of diagnosis. We found retrospective data searches easily identified these patients. Collaboration and integration of genetics services and cancer services ensured a broader spectrum of patients were included. Further consideration will be needed to examine how we can integrate routine quality assurance projects across specialties in our cancer centres to improve and update genetic information for patients within our cancer centres.

### Strengths and limitations

The use of the Trakgene genetic database was a strength in this study, as it provided information on referrals across the whole state. Obtaining records from the electronic medical records was also a strength. Across both databases data entry and retention were limitations of this study. There were some inconsistencies in the data entry across both databases, which may have resulted in some women being omitted from our data searches. Where possible information was verified by manual searches, but in some cases information was unable to be verified and that individual removed from the data set.

The retrospective nature of this study was a limitation, as the testing arranged for women in this study was not all the same, but reliant on what testing was available and routine practice in the genetics services at the time. This is particularly salient with the panel testing described in this study. The small data set was also a limitation, this study could be replicated state or nation-wide to provide greater depth to the knowledge about referral rates for women with TNBC and the outcomes of their genetic testing.

## Conclusion

We have shown referral pathways for women diagnosed with TNBC to regional cancer genetics services is working well within our cancer centres. We have identified areas of improvement which need further examination, which we think can be improved by conducting routine cross-discipline quality assurance projects. By using retrospective data searches we have been able to identify patients who may benefit from an updated genetic assessment, improving patient care in our services.

## Data Availability

The datasets used and analysed during the current study are available from the corresponding author on reasonable request.

## Abbreviations

MDT: Multidisciplinary Team
PV: Pathogenic Variant
TNBC: Triple Negative Breast Cancer
